# Protocol Deviations: A Holistic Approach from Defining to Reporting

**DOI:** 10.1007/s43441-021-00269-w

**Published:** 2021-03-29

**Authors:** Laura Galuchie, Catherine Stewart, Frank Meloni

**Affiliations:** 1 Global Clinical Trial Operations, Merck Research Laboratories, 126 East Lincoln Ave, Rahway, NJ 07065 USA; 2 Clinical Development & Operations, Pfizer R&D UK Ltd, Sandwich, Kent, UK; 3 Regulatory Medical Writing, Janssen Research & Development, Spring House, PA USA

**Keywords:** Protocol deviation (PD), Quality Management System (QMS), Risk management, Issue management, Risk assessment categorization tool (RACT), TransCelerate

## Abstract

Improving interpretation of existing guidelines and management of protocol deviation processes could increase process efficiencies and help reduce noise to support rapid identification of *important* protocol deviations. Towards this end, TransCelerate identified key principles to build upon and clarify the definition of a protocol deviation and developed a holistic approach to protocol deviation management. The approaches are flexible to suit a variety of indications, study designs, and investigational agents while also supporting consistent application within a study, program or organization.

## Introduction

Clinical study protocols are conducted according to the International Council for Harmonization guidance on Good Clinical Practice (GCP) [1] which outlines safeguards for the rights, safety and well-being of participants. Protocols “should [also] be designed, conducted and analyzed according to sound scientific principles to achieve their objectives; and should be reported appropriately” [2] If conducted as designed, the data produced should be reliable and reproducible, supporting a clear interpretation of results while protecting participants. It seems intuitive that deviations to the protocol could negatively impact the participant or interpretability of the data and should be avoided.

Nonetheless, despite efforts to minimize them, protocol deviations (PDs) do occur. They do not all have the same impact and the importance of the deviation needs to be assessed. Examples of *important* PDs, defined as those with the most impact, were provided in ICH E3 in 1996 [3]. A formal definition and additional examples were provided in ICH E3 Q&A R1 in 2012 [4]. Despite a formal definition with examples, different interpretations of importance exist [5, 6].

Over-interpretation of this definition may lead to the inclusion of situations which are not PDs, such as theoretical situations. The addition of these extraneous situations could potentially delay identification of important patient safety information by increasing noise in the system. Under-interpretation may exclude situations based on fault or other reasons and could decrease the reliability of study results related to both effectiveness and safety. This range of interpretation contributes to varied and sometimes conflicting instruction to sites. This limits their ability to identify PDs and establish preventative actions which may result in direct impact to participants. It may also delay reporting or obscure interpretation of PDs by institutional review boards (IRBs) or ethics committees (ECs).

## Methods

Subject matter experts from TransCelerate Member Companies formed a cross-functional working group to understand common challenges related to PDs. To confirm and further articulate issues, the team conducted blind surveys and interviews and reviewed literature and multiple guidelines. A common factor seen across stakeholders was variation in the definition of important PDs. In response, the team developed key principles building upon existing definition and incorporated ICH E6 R2 elements and associated methodology. Health authority comments and questions were addressed, resulting in a toolkit that was posted for public comment. Industry representatives were informed of a public comment period via conference presentations [7–9] and webinars [10, 11]. Public comments were incorporated into the toolkit.

## Results and discussion

Key principles were developed to build upon and clarify the definition of a PD. However, applying a refined PD definition is not a one-time effort. To guide sponsors, CROs and investigational sites in identification and management of PDs, a toolkit was developed. It incorporates risk-based approaches from ICH E6 R2 and risk and issue management concepts [12] from the TransCelerate clinical Quality Management System conceptual framework [13]. The toolkit components are designed to be used together, but the approaches are flexible to meet the needs of study designs and stakeholders. Examples provided are not intended to be all-inclusive, exhaustive, or mandatory.

**Fig. 1:**
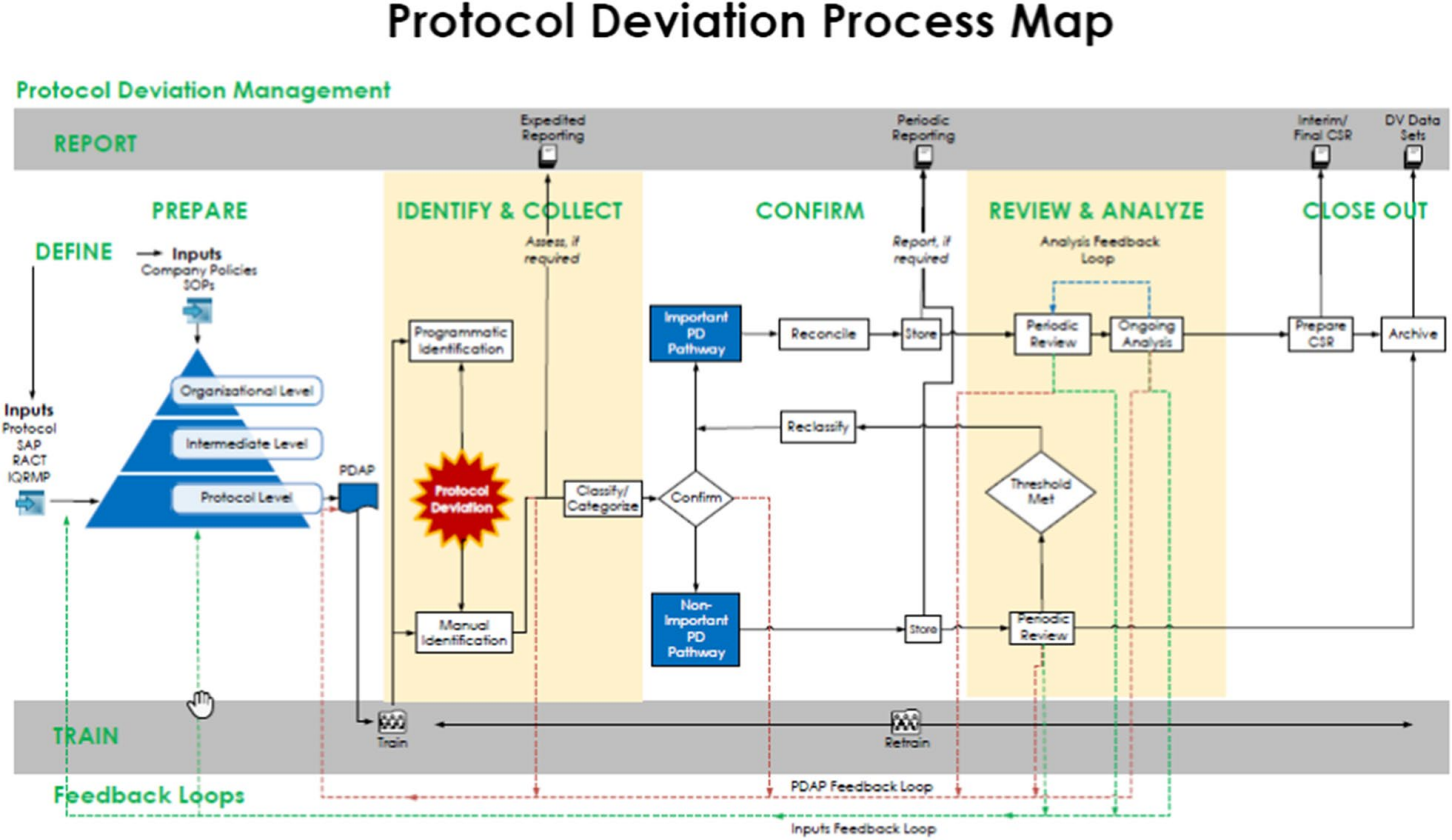
Protocol deviation map

The Protocol Deviation Process Map (Fig. 1) illustrates a holistic approach to management of PDs. Process steps are repeated throughout the clinical study as emphasized by the feedback loops and ongoing activities, which are illustrated as horizontal bars.

### Define

ICH E3 Q&A R1 defines a PD as “…any change, divergence, or departure from the study design or procedures defined in the protocol.” This definition is often over-interpreted leading to inclusion of a wide scope of items such as theoretical situations, and situations which are not PDs. For example, discovery that training of a Clinical Research Associate (CRA) was delayed needs to be addressed, but it is not a PD. This wide scope of items generates noise and could delay identification of trends or dilute impact of actual PDs.

For these reasons, we recommend the following clarifying key principles:

1. An event occurred (e.g., not theoretical)
2. The event is related to the protocol or documents referenced in the protocol (e.g., laboratory manual)
3. The event is independent of fault, blame or circumstance – to ensure an objective approach to identification (e.g., sample tube broke en route to central laboratory)

Events, issues or situations that are not PDs may require action or follow-up through other processes.

Once a PD is identified, it can then be categorized as important or non-important. Risk-based approaches from ICH E6 R2 can be applied to the definition of important PDs. The updated definition becomes:

*“Important protocol deviations* are a subset of protocol deviations that may significantly impact the completeness, accuracy, and/or reliability of **key** study data or that may significantly affect a subject's rights, safety, or well-being. For example, important PDs may include enrolling subjects in violation of key eligibility criteria or failing to collect data necessary to interpret primary endpoints, as this may compromise the scientific value of the trial.”

ICH E3 Q&A R1 indicates sponsors have some flexibility in determining what is an important PD, stating the “definition of important PDs for a particular trial is determined in part by study design, the critical procedures, study data, subject protections described in the protocol, and the planned analyses of study data.” Building on this guidance we suggest the following interpretations:

- The term “protocol deviation” is preferred over the term “protocol violation.” Local HA and IRB/EC may have other specific definitions;
- “Significant” in the context of PDs is not a statistical term;
- “Important,” “major,” “critical” and “significant” are synonyms when referring to important PDs.

Moving forward, use of “important” is proposed as common terminology.

The concepts of key or critical study data and processes are not new. They were outlined in the 2011 draft and 2013 issuance of FDA’s Guidance for Industry “Oversight of Clinical Investigators – A Risk-Based Approach Monitoring” [14] as well as the 2016 ICH E6 R2. They continue to be key component of risk-based approaches to clinical study management [15]. We believe the same risk-based principles apply to defining important PDs.

ICH E3 does not provide a formal definition of a non-important PD. It appears reasonable, however, to consider a PD non-important if it does not meet the criteria for important. Both important and non-important PDs are collected, processed and reported, although the pathways may differ as discussed later.

**Fig. 2:**
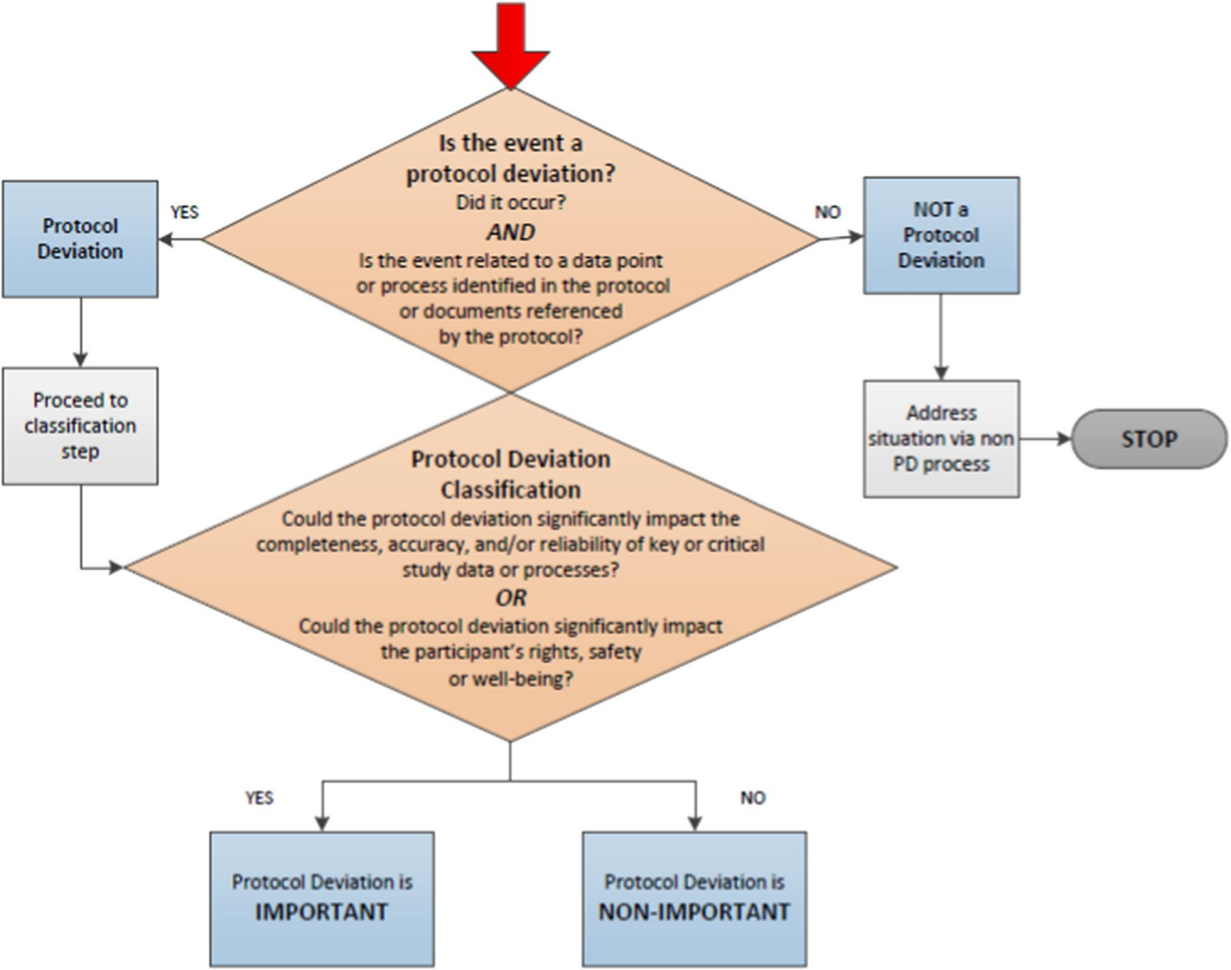
Protocol deviation decision tree.

The PD Decision Tree (Fig. 2) can be applied to support consistent application of critical thinking within an organization, and potentially across the industry. Conducting periodic therapeutic area or indication reviews is a best practice to maintain consistency within a company.

### GCP Compliance

Clinical studies are conducted according to GCP. Protocols make direct reference to GCP, and some have assumed all GCP compliance issues are also PDs, thus inflating the volume of events. For example, a missing signature on the Delegation of Authority log needs to be addressed, perhaps as an action item. In most cases, this granular procedure is not written in the protocol and is not a PD.

To reduce noise generated by these types of events, we propose to address GCP issues outside the PD process unless they meet the classification of *important* as outlined via the PD Decision Tree (Fig. 2).

Examples of GCP compliance issues which may also be important PDs include:

- Study participant received expired investigational product
- Key or critical study procedures performed by study site staff without appropriate qualifications or training

Some GCP compliance issues may also qualify for expedited reporting to Regulatory Authorities depending on local regulatory requirements (e.g., serious breaches). Companies should follow their escalation and assessment paths for decision making and reporting.

### Protocol

The protocol and documents referenced in the protocol are the primary sources when determining whether something is or is not a PD. Therefore, a best practice is to conduct risk assessment reviews and define PD classification approaches prior to protocol finalization. This allows for changes to reduce occurrence of PDs.

### Prepare

**Fig. 3:**
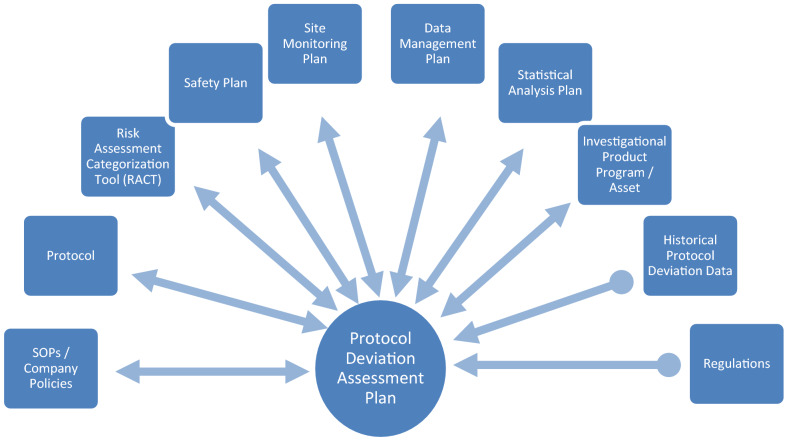
Relationship between protocol deviation assessment plan (PDAP), quality and risk management plans and other documents

A protocol-specific protocol deviation assessment plan (PDAP) documenting the management of PDs is recommended to support consistency within a study, across a program, and within a company or organization. As illustrated in Fig. 3, it may be created as a stand-alone document or incorporated into existing quality and risk management plans. Whatever the form, we recommend creation in conjunction with protocol development and maintenance as a living document, with suitable version control measures, until the last study data has been reviewed.

**Fig. 4:**
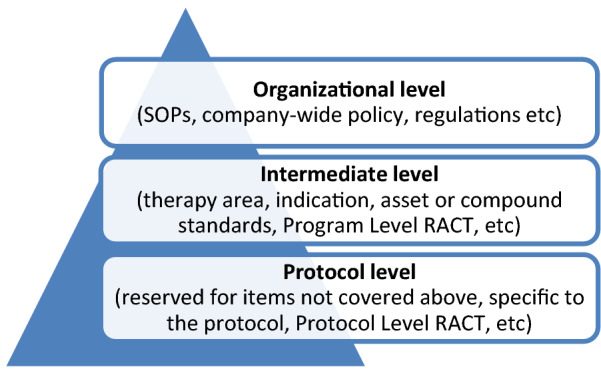
PDAP levels: organizational, intermediate, protocol

The first step is to define and prospectively identify important PDs which may occur. Inputs may come from organizational, intermediate and protocol level components as illustrated in Fig. 4. Although all three inputs are used, we recommend use of organizational and intermediate level definitions whenever possible, augmented with protocol level definitions where necessary. This will reduce variability in classification and categorization and help support consistency in analysis and reporting.

**Fig. 5:**
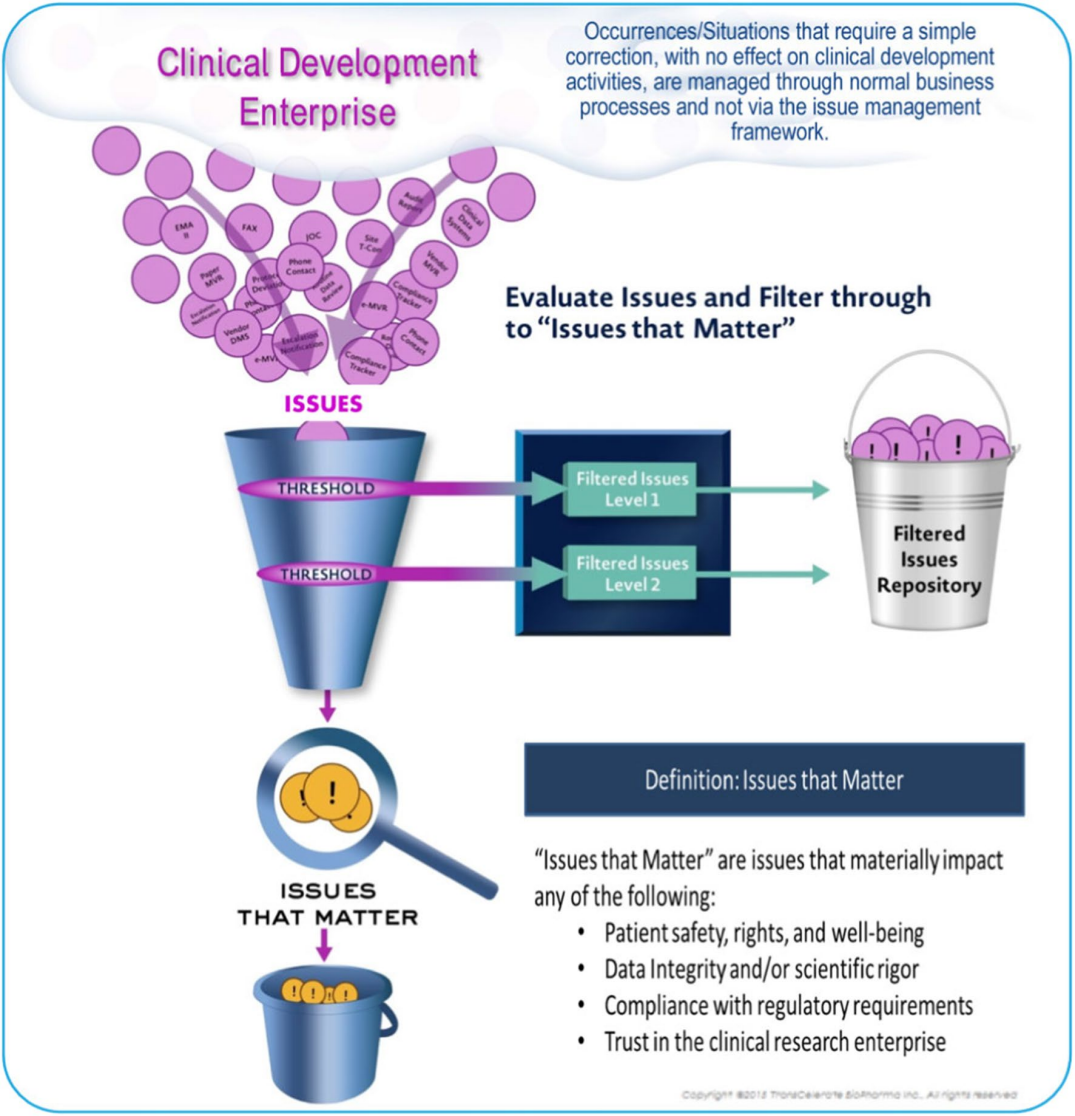
Issue management illustration

Use of an issue management approach supports consistent identification, classification and categorization of PDs (Fig. 5) and consistent responses to the question “What is an important PD?”.

Finally, teams may find program-level or protocol level risk assessment tools useful to identify potential situations which would be considered important PDs.

Under this proposed approach, the PDAP should describe PDs classified as important. A pragmatic approach should be applied when describing non-important PDs, for example, only include those commonly misclassified or part of a classification threshold.

The PDAP template includes the following recommended elements:

- Guidance for consistent classification and categorization of PDs,
- Method of identification for potential important PDs and primary team members involved,
- Thresholds at which non-important PDs may become important.

Additional elements may be included:

- Frequency of data reviews or trend analyses,
- Feedback and/or escalation pathways,
- Type and extent of reconciliation (e.g., between PD collection tool and the clinical database),
- Documentation, approval, and archiving requirements.

The PDAP is a living document and is intended to be reviewed and updated throughout the study. It should remain a living document until the last study data has been reviewed. The PD Decision Tree (Fig. 2) can also be used during study conduct.

### Train

Training should be provided to relevant study team members, emphasizing their role. As a best practice, the following approach is recommended:

- Train study site personnel on the protocol including amendments, not the PDAP,
- Train study team (including CRAs, CROs, vendor, etc.) on the protocol including amendments and the PDAP including updates.

Retraining and feedback mechanisms should also be implemented.

### Identify and Collect

PDs can be identified via programmatic or manual processes. Programmatic identification is based on data captured in a database using an electronic/computerized process or program. Manual identification relies on human interpretation. In both cases, a well-defined PDAP is essential for consistency and timely identification of PDs. Describing the method of identification of potential important PDs ensures alignment of efforts and focuses team members on items which cannot be identified via the alternate approach.

Manual identification approaches can vary but should focus on information not captured in an electronic system. As a best practice, identification of potential PDs should rely on programming and reports whenever possible. The identification of those elements which are not programmable should be a primary focus for CRAs during on-site visits.

Application of risk-based monitoring methodology may not identify *all* PDs. However, it should identify *important* PDs. Risk assessment activities identify critical data and processes that matter most in the clinical study. Armed with this prioritized information, the study team can focus activities on PDs with the greatest impact on participant safety and reliability of study data.

If important PDs are identified during study conduct, which are not included in the PDAP, the study team should consider updating the PDAP.

### Classify and Categorize

Each PD should be classified and categorized upon identification, ideally by the identifier.

- Classify is defined as determining if the PD is important or non-important.
- Categorize is defined as the type of PD (e.g., inclusion/exclusion).

The classification of a non-important deviation can change. Triggers for reclassification may include:

- meeting a pre-determined threshold
- increase in volume, frequency or cadence

Conversely, PDs classified as important may be reclassified to non-important.

The PDAP template contains four categories from ICH E3 plus three additional recommended categories. Table 1 in Appendix contains examples of classification and categorization to guide in identifying situations which are important, non-important and not a PD.

### Confirm

PD classification and categorization should be confirmed. It is recommended that an independent study team member (or group), other than the identifier, perform this review. Feedback and possible retraining should be provided to the identifier if the PD is misclassified, miscategorized, or otherwise erroneously reported.

Based on collection method (programmatic and/or manual), **important** PDs should be reconciled, and all discrepancies should be addressed. Reconciliation may include:

- removal of duplicate PDs (e.g., those identified via both programmatic and manual methods)
- consistency between data point(s) and PD (e.g., transcription error corrected eliminating the PD)

For **non-important** PDs, periodic aggregate reviews should be completed to identify trends or systemic errors which may meet a threshold to upgrade the classification to important.

### Store

PDs, including the classification and categorization and any associated data points, should be stored in a validated repository or system to support review and reporting (e.g., Clinical Trial Management System [CTMS], Electronic Data Capture [EDC], Trial Master File [TMF] or a custom system).

Key elements for storage considerations are the ability to retrieve or regenerate both important and non-important PDs for various analyses and reporting needs.

The PDAP should be stored with other protocol related decision-making records.

### Review and Analyze

Study teams typically conduct periodic reviews, monitor clinical study data and conduct analyses during study conduct. The results may be leveraged to identify important PDs not previously included in the PDAP and to assess whether frequency or volume of non-important PDs should trigger reclassification. If existing activities cannot be leveraged, additional efforts should be considered.

Important PDs are one of several factors used to determine which participants are excluded from the “per protocol” analysis study population pre-specified in the Statistical Analysis Plan (SAP). Impact of important PDs should be discussed in the CSR.

### Report

Periodic reporting during the clinical study to a central IRB, local IRBs, EC, and/or Health Authorities varies on cadence and content. Both important and non-important PDs should be reported as required.

Guidance for discussing important PDs is addressed in ICH E3. The below recommendations apply to Section 10.2 for both interim and final CSRs.

- Include a high-level, study-specific summary of *important* PDs that occurred. Discuss impact on participant safety or interpretation of study results. The impact may be by participant level, investigational site level or overall.
- Display number of participants whose important PD(s) resulted in exclusion of any/all of their data from any/all analyses.
- Summarize important PDs by category.

GCP issues are usually described in a separate section of the CSR. However, Section 10.2 may include a reference to those participants whose important PDs resulted from a GCP issue(s).

### Close Out

Important PDs are summarized and included in the CSR and archived. Non-important PDs should be archived in a validated repository or system to support future reviews and PD data sets (e.g., SDTM). Additionally, protocol level important PDs may be considered for use at intermediate or organizational levels.

## Conclusion

Stakeholders, including sites and CROs, reported frustration, inefficiencies and challenges in multiple areas related to identification and processing of important PDs that could lead to missed issues and create potential patient safety concerns. The variety of definition interpretations was identified as a common factor. Key principles were developed to clarify what constitutes a PD and risk-based approaches from ICH E6 R2 were applied to the definition of an important PD.

A toolkit was developed to support a holistic approach in management of PDs. The tools are flexible to suit a wide variety of indications, study designs, and investigational agents while also providing tools for consistent application within a study, program or organization.

Together, building on the definition of an important PD and using fit-for-purpose tools will support stakeholders for their role in rapid identification and management of important PDs which have the most impact on patient safety or data integrity.

## Supplemental Resources

Toolkit components are available for download from TransCelerate’s website [16]. Resources for Common Protocol Templates [17], Quality Management Systems [18] and Risk Based Monitoring [19] are also available.

## Appendix

**Table 1:** Protocol deviation classification and categorization examples

Category	Protocol deviation classification examples		
	Important	Non-important	Not a protocol deviation
Informed consent	• Clinical study procedures conducted prior to obtaining initial informed consent • New clinical study procedures performed before participant was re-consented • Re-consent containing updated risk language or important safety information not signed	• If required by local regulation or IRB/EC, participant did not initial all pages	• Administrative items such as: participant did not use requested date format, participant did not sign on requested line, etc
Inclusion/exclusion	• Participant entered the clinical study without satisfying entry criteria		
Study intervention	• Participant received wrong study treatment • Participant received the incorrect dose unit, route of administration, and/or inaccurate frequency of administration or expired product • Participant was non-compliant with study medication/treatment (e.g., above or below protocol-specified threshold, overdose) • Participant received and took study medication which underwent a temperature excursion and was deemed unacceptable for use	• Participant was dispensed study medication which underwent a temperature excursion and was not taken or was taken but deemed acceptable prior to use • Stratification error/missed stratification	• Investigational product underwent a temperature excursion but was never dispensed to a participant • NOTE: May be considered a GCP issue for resolution outside of PD process • Investigational product had a temperature excursion which was determined to be within acceptable range before it was provided to a participant
Prohibited concomitant medication	• Participant took an excluded concomitant medication during the clinical study • Participant took a specific class of medication within X days before a specific procedure [outside the window, taking the medication may be considered non-important]	• Instructional text for windows of analysis in the protocol may result in non-important deviations • Participant took a single dose of a class of medication [repeated use may be considered important]	
Trial procedures	• Missed safety or efficacy assessments related to primary or key secondary endpoints • Key safety or efficacy endpoint data collected on equipment which was not properly calibrated at protocol defined time points • Specific personnel for key or critical protocol specific procedures did not complete specific training (e.g., in a neuroscience therapy area, the rater was not trained on how to assess a key study endpoint)	• Procedures not directly related to participant safety (e.g., outcomes research) • Repeat efficacy measures not performed after predefined endpoints • Missed procedures that have no impact on reliability of study results (e.g., exploratory analysis) • Missed laboratory measurements that are not part of key or critical safety or efficacy endpoints • Non-critical procedures performed out of a specified window • Failure to calibrate equipment relating to non-key safety or efficacy endpoints, at protocol defined time points	• Anticipated quantity of lab collection kits not on-site • Not calibrating a piece of equipment on a day it was not used to obtain participant data • Training of CRAs or other sponsor personnel • Note: In general, training is not a PD. It is an issue that does need corrective action and appropriate follow-up
Safety reporting	• Serious Adverse Events (SAEs) or Pregnancy not reported within required reporting timeframe (e.g. 24 h from awareness) • Events of Special Interest (e.g., potential drug induced liver injury [DILI], Hy's Law, major adverse cardiac event) not reported within protocol specified timeframe	• Non-serious AEs (NSAEs) not reported within predefined protocol timelines	• Site appropriately reported an SAE. Later, the sponsor data management team asked for the SAE to be split and recorded as multiple events. The time stamp of the new data entry made it appear that the site was delayed, but they were not
Discontinuation	• Participant developed withdrawal criteria during the clinical study but was not withdrawn • Participant developed withdrawal criteria for study treatment but was not withdrawn from study treatment		

This table offers examples of PD classification and categorization to guide stakeholders in defining important and non-important PDs including ICH E3 Guideline examples. This list is not intended to be all-inclusive or exhaustive. All users are responsible for ensuring compliance with relevant laws and regulations
